# The Relationship between Abdominal Diastasis and Lumbar Pain Pressure Threshold in Women Who Have Given Birth between the Ages of 30 and 45 Years—An Observational Pilot Study

**DOI:** 10.3390/medicina60040591

**Published:** 2024-04-03

**Authors:** Ana González-Muñoz, Leo Pruimboom, Santiago Navarro-Ledesma

**Affiliations:** 1 Clinical Medicine and Public Health PhD Program, Faculty of Health Sciences, University of Granada, Av. de la Ilustración, 60, 18071 Granada, Spain; 2 Clinica Ana Gonzalez, Avenida Hernan Nuñez de Toledo 6, 29018 Malaga, Spain; 3 Chair in Clinical Psychoneuroimmunology, Faculty of Health Sciences, Campus of Melilla, University of Granada and PNI Europe, 2518 JP The Hague, The Netherlands; leo@cpnieurope.com (L.P.); snl@ugr.es (S.N.-L.); 4 Department of Physiotherapy, Faculty of Health Sciences, Campus of Melilla, University of Granada, Querol Street, 5, 52004 Melilla, Spain

**Keywords:** diastasis, inter-rectus diastasis, low back pain, pain pressure threshold, ultrasound

## Abstract

*Background and Objectives*: Current evidence confirms that the magnitude of the inter-rectus distance (IRD) is associated with the severity of abdominal pain. Furthermore, evidence exists in the literature about the impact abdominal muscles have on low back pain, lumbopelvic pain, breathing and lumbar abdominal strength; however, no studies analysing the level of association between abdominal diastasis and lumbar pain pressure threshold (PPT) exist. The aim of this study was to analyse the level of association between the rectus abdominis distance and pain pressure threshold in the lumbar spinous processes in women who have given birth between the ages of 30 and 45 years. Secondly, it was to study the level of association between the time elapsed since the last delivery and low back pain in women who have given birth between 30 and 45 years of age. *Material and Methods*: This was a pilot observational study in which 21 females participated. The abdominal diastasis was measured by ultrasound, the pain pressure threshold was assessed by an algometer and the pain perception by the Mc Gill questionnaire. *Results*: There was no significant relationship between increased abdominal distance and increased lumbopelvic pain in women who gave birth between the ages of 30 and 45 years. However, there was a correlation between the time that had elapsed since the last delivery and low back pain. *Conclusions*: there was a correlation between the time that had elapsed since the last delivery and low back pain. Further studies analysing factors that may perpetuate the chronicity of symptoms, such as lifestyle and intrinsic factors, are needed.

## 1. Introduction

Abdominal diastasis (AD) is defined as the separation of the rectus abdominis muscles along the linea alba (LA), accompanied by a detriment in the quality of this tissue [1,2,3,4]. It is a very common condition in pregnancy, with an incidence of 66% in the third trimester, which then persists in the immediate postpartum period in 35–60% of women, and in 36%, it continues for up to 12 months after delivery [1,2,5]. Between 25 and 45% of women experience low back pain during pregnancy and the immediate postpartum period [6]. Pelvic pain, which is pain commonly reported between the posterior iliac crest and the gluteal fold, is suffered by 20–25% of pregnant women [7]. After pregnancy, the persistence of pain remains in 15–45% within the first 6-month postpartum period, with up to 51% of these women continuing to experience pain even 3 years after childbirth [6,7,8].

There are a number of factors, such as abdominal obesity, significant weight loss or weight gain in a short period of time, diseases that affect the formation of collagen, frequently performing activities that produce abdominal hyper-pressure and soft tissue degeneration, that increase the likelihood of suffering from AD [4,9]. Multiparity, multiple pregnancies, caesarean sections, maternal age over 35 years or high newborn weight are specific characteristics that also predispose pregnant and postpartum women to AD [2,10,11,12]. In addition to these predisposing factors, dysfunctions such as prolapses, urinary or faecal incontinence or abdominal hernias that result from inter-rectus separation and affect the lumbopelvic abdominal area also exist [4,9], as does an unsatisfactory body image and the consequent problems that it has on patients’ self-esteem [1,2,12,13,14].

Currently, the assessment of AD is carried out using different procedures such as a measuring tape to measure between one rectus abdominis muscle and another externally, in addition to magnetic resonance imaging and ultrasound [3,4]. Due to its quick assessment, accuracy and the low cost of its use, ultrasound is considered the technique of choice for the assessment and diagnosis of AD, being the gold standard [9,15,16]. It has shown that a value of up to 1.5 cm between one rectus abdominis muscle and another is considered normal, while more than 2 cm between the recti is considered AD [4,9]. Additionally, ultrasound can provide information about muscle thickness in different muscle groups in both a state of activation and at rest; in this case, the thoracoabdominal girdle [17,18] will be one of the structures under assessment in this study.

The lumbar musculature is characterised by its stabilising function in relation to the pelvis; therefore, an increase in the distance between the rectus abdominis muscles outside the physiological limits affects the stability of the lumbar area. This is the reason why patients with AD are more likely to suffer from low back pain, pelvic pain and lumbopelvic pain compared to healthy subjects [1,2,12,13,14,19].

During pregnancy, the greatest disturbance in the arrangement of these structures is produced [3]. One of the hormones responsible for this is relaxin, which is secreted during the gestational period. This changes the composition of collagen and favours elasticity and laxity, resulting in the abdominal wall and other structures that are rich in connective tissue becoming more susceptible to mechanical stretching [2,4]. The overstretching of the abdominal wall produced by the growth of the foetus will more or less produce inter-rectus separation [20] depending on various factors, which include the mother’s genetics in terms of the quality of LA connective tissue, the two rectus abdominis muscles, and the weight and size of the foetus, or the quality of the abdominal girdle in regard to its previous preparation or training [2,4,18]. This inter-rectus separation alters the spatial arrangement of the rectus abdominis muscles and their fascia, resulting in a modification of their optimal line of action and reducing their ability to generate force and offer resistance [4,13,17,21].

The evolution of acute lumbopelvic pain into chronic pain is complex and further research is needed to clarify why some women develop long-term lumbopelvic pain after childbirth, why some are at greater risk than others and whether its prevention is possible [5]. Although most women recover from these symptoms, on average, within 6 months, recent studies have shown that lumbopelvic pain can persist for 2 to 12 years postpartum [22].

In addition to the aforementioned biomechanical factors, recent advances in neuroscience have tried to correlate the greater predisposition to suffering low back pain during pregnancy and postpartum [2,6,12]. The main causes of low back pain are degenerative disk disease and changes in vertebral plates.

Oestrogen and progesterone hormone receptors are present in vertebral plate cartilage tissue; hence, any change in the rate of hormone release during pregnancy influences the risk of developing disk and vertebral body degeneration, and consequently, lumbopelvic pain [23].

Algometry is defined as a measurement method that quantitatively evaluates the pain pressure threshold (PPT) that a patient refers to [24,25]. It is carried out using an algometer, which is a device that accurately measures the pressure applied to an anatomical area of choice, and allows for a relationship to be established between the presence of pain and the functionality of a structure [24,25,26].

Studies confirm that the magnitude of the inter-rectus distance (IRD) is associated with the severity of abdominal pain [19,27]. Also, evidence exists in the literature about the impact abdominal muscles have on low back pain, lumbopelvic pain, breathing and abdominal strength [2]. The lumbar musculature is affected when the distance between the rectus abdominis muscles is outside the physiological limits and this facilitates suffering from chronic low back pain [1,2,12,13,14,19]. The involvement of the abdominal myofascial system is very important in chronic pelvic pain due to the presence of myofascial trigger points that represent irritable points located inside the fascia, in this case the abdominal fascia, which explains that women with chronic pelvic pain caused by myofascial syndrome obtain lower results in the PPT measured by algometry [5].

The hypothesis of this study was that there is a relationship between the rectus abdominis distance and low back pain in women who have given birth between the ages of 30 and 45 years. To the best of our knowledge, there are no studies analysing the level of association between abdominal diastasis and PPT in the low back [12,13]. Thus, increasing knowledge in this regard would be of great value to the clinical field [13]. It is worth noting the relationship existing between lumbar PPT and abdominal diastasis due to the structural relationship presented at the myofascial and bone tissues. The increase in abdominal diastasis may generate a decrease in abdominal–lumbopelvic motor control, and that may be aggravated if there is a lack of tone in the main lumbopelvic stabilising muscles, i.e., the transversus abdominis. This context would generate an excess of tension and traction in the lower back when hypertensive postures, which can trigger the activation of lumbar myofascial trigger points and therefore irritation of the corresponding metameric levels.

Therefore, the goal of this study was to analyse the level of association between the rectus abdominis distance and pain pressure threshold in the lumbar spinous processes in women who have given birth between the ages of 30 and 45 years. Secondly, it was to study the level of association between the time elapsed since the last delivery and low back pain in women who have given birth between 30 and 45 years of age.

## 2. Materials and Methods

### 2.1. Study Design

This was a pilot observational study of female participants from the province of Malaga, who met the required inclusion criteria. The assessment protocol followed in this study was designed in accordance with the fundamental principles for clinical research in humans described in the Declaration of Helsinki. The study was approved by the Ethics Committee of the European University of Madrid (NR21911981), Spain, and is reported in line with the standard protocol elements: Statement of the Recommendations for Intervention Trials (STROBE) [28].

### 2.2. Participants

A total of 25 women aged between 30 and 45 years were recruited for the study by conveniently sampling those volunteers who wanted to participate. The participants were primiparous (four participants) and multiparous (twenty-one participants); that is, they had had one or more children, respectively. All of the subjects gave their written informed consent, in addition to receiving information about the characteristics of the study before having any measurements taken.

The patients were recruited and evaluated at a private clinic, Clínica Ana González, which is located in Malaga, Spain. One of the authors of this present work was in charge of taking the measurements (AGMs).

The inclusion and exclusion criteria for participation in the study were as follows.

Inclusion criteria:

1. Subjects between 30 and 45 years of age;
2. Subjects had one or more children.

Exclusion criteria:

1. Systemic autoimmune diseases such as rheumatoid arthritis;
2. Neurodegenerative diseases;
3. Neurological diseases;
4. Tumoral processes;
5. Physical or cognitive inability to respond to the demands and needs of the study;
6. Febrile state due to a viral or infectious process;
7. Pregnant women;
8. Previous spinal surgeries or fractures.

### 2.3. Variables

#### 2.3.1. Demographic Variables

The sociodemographic characteristics of the participants were collected by means of a data collection sheet detailing the following: age, weight, height, body mass index (BMI), number of deliveries, type of delivery (vaginal or caesarean), time since delivery and epidural (measured in epidural numbers according to the number of deliveries).

#### 2.3.2. Outcome Measures

Every patient had a private appointment at the aforementioned clinic. Firstly, they were given the information sheet which detailed the study, followed by the participants signing the informed consent form. The researcher also collected the following data: age, height, weight, the BMI (previously calculated), the number of deliveries, the type of delivery (vaginal or caesarean), whether or not they had an epidural and the number of years since their last delivery [21].

##### Distance between Rectus Abdominis Muscles

The inter-rectos distance was assessed via ultrasound using a Sonosite M-turbo (GE Healthcare, Wauwatosa, WI, USA) ultrasound machine. The participants laid in a supine position on the bed with the abdominal region undraped. The images were taken using a 6–13 megahertz (MHz) linear transductor with a width of 6 cm.

The ultrasound parameters were adjusted to optimise the quality of the image. Likewise, the depth was adjusted to clearly view the rectus abdominis and the linea alba muscles of the subjects.

**Figure 1:**
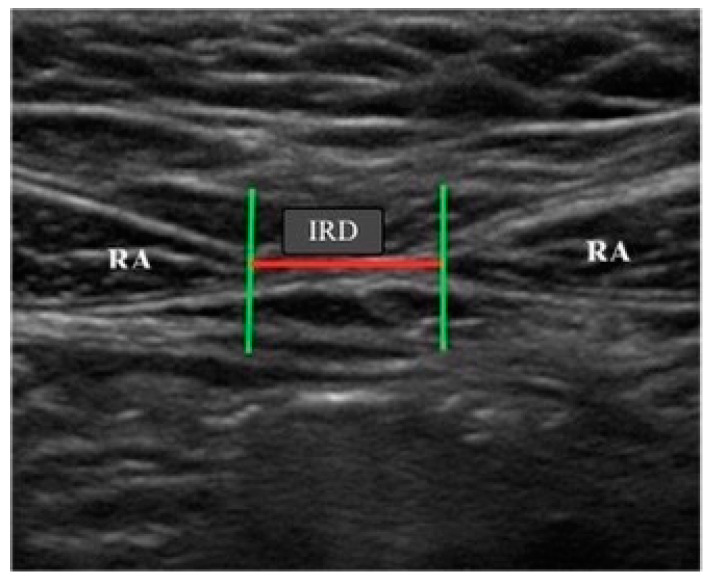
View of the rectus abdominis muscles and measurement of the inter-rectus distance (IRD).

The transductor was placed perpendicular to the abdominal surface at three different points: 2 cm above the navel, 2 cm below the navel and 5 cm above the navel. The inter-rectus distance (IRD), in centimetres, was measured at each of these points [18,29] (Figure 1). All the measurements were carried out by the same physiotherapist who specialised in using this equipment.

##### Pain Pressure Threshold of the Thoracic and Lumbar Spinous Processes

The pain pressure threshold of the thoracic and lumbar spinous processes was assessed by algometry using a Wagner analogue algometer. A PPT training protocol to increase the quality of the PPT assessments was carried by the physiotherapist in charge of the PPT measurements two weeks before the beginning of the study. A standard pressure algometer (FPK 20; Wagner Instruments, Greenwich, CT, USA) exerting up to 4 kg of pressure on 1 cm^2^ was used to assess each lumbar spinous process, from T5 to L5 [30].

Participants were placed in a prone position with the upper limbs extended. The algometer was placed perpendicularly to the bony relief of the spinous processes from T5 to L5, with continually increasing pressure until pain was perceived by the patient. Three measurements were carried out for each point followed by the calculation of the average. The measuring process ended at the moment when the patient felt an increase in sensitivity [24,25].

##### Intensity of Low Back Pain

The assessment of pain intensity was conducted utilising the McGill Pain Questionnaire, encompassing various pain descriptors and employing a scoring system ranging from 0 to 5. This questionnaire furnishes quantitative data amenable to statistical analysis. It exhibits ample sensitivity to discern variances among distinct pain alleviation methodologies, offering insights into the relative impacts of specific interventions on the sensory, affective, and evaluative facets of pain perception. The patients completed the Spanish version of the McGill questionnaire, making reference to the current level of lumbar pain in a completely subjective way. This questionnaire assesses different areas where the patient has pain: its location, its characteristics, its intensity and rating of the current pain measured by a visual analogue scale (VAS) [31,32,33].

### 2.4. Statistical Analysis

The statistical analysis was carried out using Jamovi for Windows version 1.1.9.0. The Shapiro–Wilk test was used to assess the normality of the sample since the sample size consisted of less than 30 subjects.

Subsequently, the descriptive statistics of the whole sample were calculated, including measurements of the central tendency and its dispersion range using the mean and standard deviation (SD) to describe parametric ranges, and the median and the interquartile range to describe non-parametric data.

Finally, the Spearman correlation was used to study the association level between the different variables since they were non-parametric data. A weak correlation was defined as values between 0.3 and 0.5, moderate between 0.5 and 0.7, and strong if the correlation coefficient was more than 0.7. *p*-value < 0.05 was considered statistically significant.

A linear multivariable regression model was built to observe the potent association between the outcome measure (IRD 2 cm above navel), PPT points and McGill questionnaire total score (independent variables). The model was adjusted for age and body mass index. Changes in the adjusted R2 were estimated, as were collinearity, autocorrelation, homoscedasticity and linearity through the correlation matrix, Durbin–Watson’s coefficient, tolerance, variance inflation factor and analysis of residuals. *p*-value < 0.05 was considered statistically significant.

### 2.5. Sample Size Calculation

The anticipated mean disparity between the intervention and placebo cohorts is envisaged to be 2 points on the Numeric Pain Rating Scale (NPRS), a magnitude considered clinically significant. Accounting for a standard deviation of 2.0 units on the NPRS, a significance threshold (α) of 0.05 and a statistical potency of 90%, a minimum sample size of 22 patients per group was deemed necessary to effectively discern the differences between the cohorts [34]. Drawing from prior investigations on pain pressure thresholds in individuals afflicted with nonspecific lumbopelvic pain, it becomes imperative to incorporate at least 18 subjects to achieve a statistical power of 80% and maintain an alpha level (α) of 0.05 [35]. The current inquiry adopted a pilot framework, analysing a singular group of participants, encompassing a total cohort of 25 subjects who actively partook in the investigation.

## 3. Results

The sample comprised 25 women with a mean weight of 69.4 ± 13 kg, height of 165 ± 6.84 cm, BMI of 25.7 ± 5.10 and number of deliveries of 2.05 ± 0.740 (See Table 1). There were no differences in the height, weight or BMI, but there were differences in the number of deliveries and the years since delivery (See Table 2) [35].

Linear regression analysis with IRD 2 cm above the navel measurement as the outcome was carried out, showing no significant associations after adjustment for age and BMI (See Table 3).

There was no correlation between the distance of the rectus abdominis muscles 2 cm below the navel and the PPT in lumbar spinous processes (L1, L2, L3, L4 and L5) in women between 30 and 45 years of age with one or more children( (r = −0.177; *p* = 0.483); (r = −0.219; *p* = 0383); (r = −0.179; *p* = 0.478); (r = −0.376; *p* = 0.124); (r = −0.338; *p* = 0.171)).

There was no correlation between the distance of the rectus abdominis muscles 2 cm above the navel and PPT in lumbar spinous processes (L1, L2, L3, L4 and L5) in women between 30 and 45 years of age with one or more children ((r = −0.037; *p* = 0.881); (r = −0.041; *p* = 0.867); (r = 0.011; *p* = 0.966); (r = −0.271; *p* = 0.261); (r = 0.123; *p* = −0.231)).

There was no correlation between the distance of the rectus abdominis muscles 5 cm above the navel and the PPT in lumbar spinous processes (L1, L2, L3, L4 and L5) in women between 30 and 45 years of age with one or more children ((r = 0.004; *p* = 0.991); (r = 0.012; *p* = 0.960); (r = 0.109; *p* = 0.657); (r = −0.012; *p* = 0.963); (r = 0.018; *p* = 0.945)).

The correlation between the elapsed time since the last delivery and low back pain in women aged between 30 and 45 years with one or more children was average (r = 0.654) and statistically significant (*p* = 0.002).

There was no correlation (r = 0.262; *p* = 0.252) between the number of deliveries and low back pain in the participants.

There was a low correlation (r = 0.454), which was statistically significant (*p* = 0.0039), between the height of the subjects and the ultrasound measurement of the IRD taken 2 cm above the navel in women who had given birth between 30 and 45 years of age.

Regarding the measurement of the different IRD levels, there was a correlation between all the measurements, showing that the correlation was high (r = 0.845) and statistically significant (*p* ≤ 0.001) between the ultrasound measurements taken 2 cm above and below the navel.

There was an average correlation (r = 0.635), which was statistically significant (*p* = 0.003), between the ultrasound measurements taken 2 cm below and 5 cm above the navel.

There was a high correlation (r = 0.850), which was statistically significant (*p* ≤ 0.001), between the ultrasound measurements 2 cm and 5 cm above the navel (See Table 4).

## 4. Discussion

This study researched the relationship between AD and PPT in lumbar spinous processes in women who had given birth aged 30 to 45 years.

The secondary objectives were to study the level of association between the time elapsed since the last delivery and low back pain in the same population, to verify the level of association between the number of deliveries and the degree of AD and low back pain, and lastly, to see if a relationship between the height and weight of the participants and the distance between the rectus abdominis muscles exists.

Consequent to completing the measurements and analysis of the data, there was no significant relation between lumbopelvic pain and an increase or decrease in AD. This result is in line with the studies by Mota et al. [1] and Sperstad et al. [11], who concluded that women with postpartum AD did not have a higher propensity to suffer from lumbopelvic pain. However, in the latter study, they acknowledged that the sample was small, and all the subjects suffered from mild AD [11].

Contrarily, Parker et al. [36] reported that women with AD tended to suffer from greater pain in the abdominopelvic and lumbopelvic regions.

More studies with a larger sample size of participants are needed to determine what the actual relationship is.

Regarding low back pain and the elapsed postpartum time, the participants’ McGill questionnaires, along with their last delivery dates, indicate that the longer the period since the last delivery, the greater the presence of low back pain (r = 0.0654; *p* = 0.002). There are many studies about postpartum low back pain in the first months after giving birth, above all, in the first year [30,37,38]; however, there are few studies that show which factors influence postpartum low back pain long-term [5]. One recent study by Bergstrom et al. [22] evaluated women with low back pain which persisted for 12 years postpartum, and subdivided them based on the psychological factors that influenced each patient’s pain. This shows the complexity of the main goal of this study, where the multifactorial origin of chronic low back pain, which affects 45% of women after delivery [6], must be taken into account, along with a biopsychosocial point of view [39,40]. Psychological factors such as an increased probability of suffering from symptoms of depression during the first three months postpartum [40,41] could be one of the factors triggering low back and lumbopelvic pain.

Algometry [24,25] of the lumbar spinous processes was used to measure low back pain in the subjects of this study. The correlation, according to the McGill questionnaire, between a longer the postpartum period since the last delivery and more low back pain experienced was not met by the algometric measurement; there was less low back pain when there was a higher pressure on the vertebral spinous processes. This lack of agreement in the data collected on low back pain has appeared before in the literature [42], namely, in a study in which both healthy subjects and subjects suffering from low back pain had measurements taken using algometry. It was shown that there was a difference between healthy subjects and those suffering from pain, but there were also differences between sensitivity to pressure and their behaviour or how each of them expressed their pain. Central sensitivity to pain and personal behaviour is often underestimated when prolonged pain is experienced [42].

Furthermore, it must be taken into account that according to this study, there was no correlation between greater low back pain and a higher number of deliveries. There are articles in the literature that define the different variables of low back pain in primipara and the factors that influence it [6,8]. However, the correlation between multiparity and the increase in low back pain is not specifically defined and is mainly related to psychological factors [5,22] such as depression or anxiety. If the influence of different psychological factors such as depression or anxiety in the presence of low back and lumbopelvic pain [1,37,39,40] is taken into account, it could explain the results of this research. Women who have experienced emotional stress during pregnancy have a higher risk of suffering from chronic lumbopelvic pain 6 months postpartum [43], with 78% of them recovering 6 months after delivery [44].

The possibility of the mother suffering from lumbopelvic pain before and during pregnancy must be taken into account, since it is a risk factor that increases the possibility of suffering it during the 14-month postpartum period [43]. One in every 10 women suffering from lumbopelvic pain during pregnancy will also suffer from lumbopelvic pain for at least 11 years after delivery, affecting their health, daily life or even economy; hence, the early detection of lumbopelvic pain symptoms during pregnancy is of paramount importance, along with proper monitoring from childbirth onwards [5]. Another study shows that women who experienced greater symptoms of depression or anxiety during pregnancy suffered from higher levels of functional disability due to greater low back pain when compared to women who presented less symptoms of depression, anxiety or low back pain [45].

We must bear in mind that in a woman who has given birth, a combination of lumbopelvic pain and depression can be overwhelming, and can compromise her daily-life activities such as standing up after spending a lot of time sitting, rolling over in bed, picking up her baby, dressing and undressing, having difficulties in her sex life due to pain, etc., reducing her quality of life considerably [2,38,43].

Relating lumbopelvic pain to AD, Deering et al. [46] observed that women with more severe AD suffered from greater lumbopelvic-stabilising muscle fatigability. This fact translates into lumbopelvic abdominal instability, since if the abdominal wall fascia is deteriorated or stretched excessively, the force generated by the abdominal muscles is reduced. In the long term, these symptoms will bring lumbopelvic pain and the risk of it becoming chronic [2,39]. More specifically, after giving birth, women show significant impairments in lumbopelvic muscle fatigability up to 26 weeks after delivery, regardless of whether the delivery was vaginal or Caesarean [46]

Finally, another result obtained from this study which coincides with the ones shown by Gitta et al. [47] and Doubkova et al. [12] is the existing correlation between an increase in IRD 2 cm above the navel and a high BMI and height (r = 0.454; *p* = 0.039). One of the facts that could explain these results is a sedentary lifestyle that leads to an increase in the BMI and, consequently, a lack of muscle mass at the abdominal level along with a significant accumulation of fat in this area. However, more studies are needed to clarify the causes.

In the same vein, when the IRD ultrasound measurement 5 cm above the navel was performed and was found to be elevated, a high IRD 2 cm above the navel was also found (r = 0.850; *p* ≤ 0.001). Mota et al. [29] found the same correlation in women assessed 6 months after delivery.

### 4.1. Strengths and Weaknesses

This study is the first to analyse the relationship between rectus abdominis distance and low back pain in women aged 30 to 45 years who have given birth. It also aimed to examine the association between the time elapsed since the last delivery and low back pain in these women. This opens new avenues for research in this area and will aid researchers and clinicians in understanding integrative postpartum treatment. However, certain limitations must be acknowledged. The pilot study design and sample size necessitate a cautious interpretation of the results, highlighting the need for future research with larger samples to corroborate the findings. Additionally, potential differences between primiparous and multiparous women may exist; thus, these differences should be considered when interpreting the results, and future research should analyse the findings in both groups.

### 4.2. Perspectives

With all the results obtained, some correlations that are not statistically significant continue to be inconclusive, the most important of which is the one between the increase in AD and the increase in lumbopelvic pain. Further studies with a larger sample of patients are necessary to clarify this. It would also be of great interest to differentiate between primiparous and multiparous women, since the vast majority of articles in the literature focus on primiparous women and their symptoms and pathologies postpartum. In this study, a correlation was observed between an increase in low back pain and the length of time after delivery. Likewise, the influence of psychological factors, such as depression and anxiety, on postpartum lumbopelvic pain and its chronicity over time was found, with pain being a personal and exclusive experience for each person. Central sensitisation to pain is a reality in patients that is often overestimated. It would be interesting to delve deeper into the multifactorial concept of pain and understand that there are many causes that influence postpartum lumbopelvic pain, and it is not only about muscular or biomechanical imbalances.

## 5. Conclusions

There was no significant relationship between increased AD and increased lumbopelvic pain in women who gave birth between the ages of 30 and 45 years. However, there was a correlation between the time that had elapsed since the last delivery and low back pain. Further studies which integrate a larger sample size and other variables, including intrinsic, psychological and lifestyle factors, are needed.

## Figures and Tables

**Table 1:** Demographic data of the sample.

	Height	Weight	BMI	Number of Deliveries	Years Since Last Delivery
Mean	165	69.4	25.7	2.05	4.25
Median	164	68	24.2	2	2.50
Standard Deviation	6.84	13.0	5.10	0.740	3.89
Shapiro–Wilk Test	0.697	0.564	0.327	<0.001	0.002

Note. BMI = Body mass index.

**Table 2:** Descriptive data of the PPT points from the T5 to L5 lumbar spinous process.

	T5	T6	T7	T8	T9	T10	T11	T12	L1	L2	L3	L4	L5
N	19	19	19	19	19	19	19	19	19	19	19	19	19
Mean	9.13	9.35	9.96	10.6	10.9	11.2	12.1	12	11.9	11.3	11.7	11.6	10.5
SD	2.89	3.82	3.55	3.73	3.65	3.43	4.18	4.21	3.94	4.21	3.74	3.87	3.61

Note. PPT = pain pressure threshold; SD = standard deviation; T = thoracic; L = lumbar.

**Table 3:** Multivariable linear regression analysis with IRD 2 cm above the navel as the outcome.

						95% CI Standardised β
Predictor	*β*	SE	*p*	Standardised β	Lower Limit	Upper Limit
Variable	5.2106	3.02	0.16			
PPT T5	1.154	0.505	0.084	2.614	−0.563	5.79
PPT T6	−1.6491	0.817	0.114	−4.944	11.747	1.86
PPT T7	−0.7033	0.568	0.284	−1.958	−6.349	2.43
PPT T8	1.0472	1.033	0.368	3.061	−5.32	11.44
PPT T9	0.239	0.646	0.73	0.685	−4.455	5.83
PPT T10	0.8413	1.196	0.521	2.264	−6.675	11.2
PPT T11	0.7202	0.746	0.389	2.363	−4.43	9.16
PPT T12	−0.6896	1.198	0.596	−2.276	13.254	8.7
PPT L1	−1.3017	0.929	0.234	−4.026	12.002	3.95
PPT L2	0.7154	0.596	0.296	2.361	−3.103	7.82
PPT L3	0.0692	0.334	0.846	0.203	−2.518	2.92
PPT L4	0.6405	0.921	0.525	1.946	−5.829	9.72
PPT L5	−0.7696	0.746	0.361	−2.182	−8.055	3.69
MCGILL	−0.2969	0.167	0.149	−1.883	−4.817	1.05

R^2^ = 0.8; Durbin–Watson’s coefficient = 2.31; VIF (variance inflation factor) = 22.4; tolerance = 0.04; PPT = pain pressure threshold; CI = confidence interval; SE = standard error; *p* = *p*-value.

**Table 4:** Correlations between the variables.

	Years Since Last Delivery	Mc Gill	Height	2 cm above Navel	2 cm below Navel	5 cm above Navel
Years since last delivery		r = 0.654 *p* = 0.002	r = −0.030 *p* = 0.900	r = 0.191 *p* = 0.421	r = 0.167 *p* = 0.495	r = 0.266 *p* = 0.256
Mc Gill	r = 0.654 *p* = 0.002		r = −0.005 *p* = 0.983	r = 0.308 *p* = 0.175	r = 0.126 *p* = 0.596	r = 0.235 *p* = 0.306
Height	r = 0.030 *p* = 0.900	r = −0.005 *p* = 0.983		r = 0.454 *p* = 0.039	r = 0.370 *p* = 0.108	r = 0.266 *p* = 0.244
2 cm above navel	r = 0.191 *p* = 0.421	r = 0.308 *p* = 0.175	r = 0.454 *p* = 0.039		r = 0.845 *p* = 0.001	r = 0.850 *p ≤* 0.01
2 cm below navel	r = 0.167 *p* = 0.495	r = 0.126 *p* = 0.596	r = 0.370 *p* = 0.108	r = 0.845 *p* = 0.001		r = 0.635 *p* = 0.003
5 cm above navel	r = 0.266 *p* = 0.256	r = 0.235 *p* = 0.306	r = 0.266 *p* = 0.244	r = 0.850 *p ≤* 0.01	r = 0.850 *p ≤* 0.001	

## Data Availability

Data are contained within the article.

## Abbreviations

DA	Abdominal diastasis
LA	Linea alba
DL	Low back pain
DP	Pelvic pain
TrA	Transverse abdominal
OI	Internal oblique
OE	External oblique
DLP	Lumbopelvic pain
IUS	Ultrasound image
UDP	Pain pressure threshold
IMC	Body mass index
IRD	Inter-rectus distance
DT	Standard deviation
RI	Interquartile range
